# Optimizing a two-layer method for hybrid diffuse correlation spectroscopy and frequency-domain diffuse optical spectroscopy cerebral measurements in adults

**DOI:** 10.1117/1.NPh.10.2.025008

**Published:** 2023-05-23

**Authors:** Rodrigo Menezes Forti, Giovani Grisotti Martins, Wesley Boehs Baker, Rickson C. Mesquita

**Affiliations:** a Children’s Hospital of Philadelphia, Division of Neurology, Philadelphia, Pennsylvania, United States; b University of Campinas, Institute of Physics, Campinas, Brazil; c Brazilian Institute of Neuroscience and Neurotechnology, Campinas, Brazil

**Keywords:** diffuse optical spectroscopy, diffuse correlation spectroscopy, multilayer model, hybrid diffuse optics, multidistance

## Abstract

**Significance:**

The sensitivity to extracerebral tissues is a well-known confounder of diffuse optics. Two-layer (2L) head models can separate cerebral signals from extracerebral artifacts, but they also carry the risk of crosstalk between fitting parameters.

**Aim:**

We aim to implement a constrained 2L head model for hybrid diffuse correlation spectroscopy (DCS) and frequency-domain diffuse optical spectroscopy (FD-DOS) data and to characterize errors in cerebral blood flow and tissue absorption with the proposed model.

**Approach:**

The algorithm uses the analytical solution of a 2L cylinder and an *a priori* extracerebral layer thickness to fit multidistance FD-DOS (0.8 to 4 cm) and DCS (0.8 and 2.5 cm) data, assuming homogeneous tissue reduced scattering. We characterized the algorithm’s accuracy for simulated data with noise generated using a 2L slab and realistic adult head geometries and for *in vitro* phantom data.

**Results:**

Our algorithm recovered the cerebral flow index with 6.3 [2.8, 13.2]% and 34 [30, 42]% (median absolute percent error [interquartile range]) for slab and head geometries, respectively. Corresponding errors in the cerebral absorption coefficient were 5.0 [3.0, 7.9]% and 4.6 [2.4, 7.2]% for the slab and head geometries and 8 [5, 12]% for our phantom experiment. Our results were minimally sensitive to second-layer scattering changes and were robust to cross-talk between fitting parameters.

**Conclusions:**

In adults, the constrained 2L algorithm promises to improve FD-DOS/DCS accuracy compared with the conventional semi-infinite approach.

## 1 Introduction

The combination of diffuse correlation spectroscopy (DCS) and frequency-domain diffuse optical spectroscopy (FD-DOS) techniques is a promising approach for continuous and noninvasive measurement of cerebral blood flow (CBF), blood oxygenation, and oxygen metabolism at the bedside.^1^^–^^3^ The approach has been validated in pediatric patient populations and swine models.^4^^–^^9^ These validation studies approximated the head as a homogeneous medium [i.e., semi-infinite (SI) head model] to derive cerebral hemodynamics from the FD-DOS/DCS signals. A well-known drawback of the homogeneous head model, however, is the significant extracerebral tissue (scalp and skull) contributions to the measured signals.^10^^–^^15^ Neglecting extracerebral contributions can result in large errors, especially for adult populations.^15^^,^^16^ Herein, we investigate the accuracy of CBF and cerebral tissue absorption coefficient measurements derived from applying a constrained two-layer (2L) head model algorithm to (a) hybrid FD-DOS and DCS simulated data in adults and (b) hybrid FD-DOS and DCS *in vitro* data in a 2L liquid phantom.

Multilayer tissue models have been investigated in prior literature separately for either DCS^12^^,^^15^^,^^17^^–^^21^ or FD-DOS data.^13^^,^^16^^,^^22^ To our knowledge, however, the simultaneous use of multilayer tissue models to fit both DCS and FD-DOS data in combination has not yet been demonstrated. Our proposed hybrid method uses a 2L cylindrical head model for simultaneous DCS and FD-DOS fitting. A cylindrical geometry is used instead of a slab geometry because the numeric approximation of the analytical cylindrical Green’s function solution for diffusive light transport is more robust.^16^ The hybrid method further incorporates the constraint of homogeneous tissue reduced scattering, which we justify below, to reduce the risk of crosstalk between unknown fitting parameters. Finally, the method fits multidistance FD-DOS (eight distances; 0.8 to 4 cm) and DCS (0.8 and 2.5 cm distances) data in sequential steps to constrain the recovery of tissue optical properties and blood flow.

To test the method, we characterized its errors across a wide range of tissue optical properties and blood flows in (1) forward-model simulations; (2) simulations in a 2L cube; and (3) simulations using a realistic head geometry. We also characterized the sensitivity of the approach to extracerebral layer thickness and DCS integration time. Finally, we performed measurements on a 2L liquid phantom to test the algorithm *in vitro*.

## 2 Methods and Materials

### 2.1 Two-Layer Head Model

**Fig. 1:**
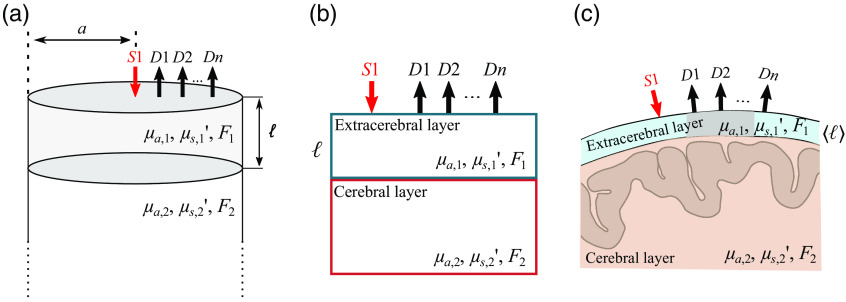
Geometry used for the 2L model and the simulations. (a) Our 2L model comprised a homogeneous cylindrical extracerebral layer of thickness ℓ and radius a (corresponding to extracerebral scalp and skull tissue) above an infinitely thick homogeneous cylindrical cerebral layer (corresponding to the brain cortex). The tissue absorption coefficient, reduced scattering coefficient, and blood flow index of the extracerebral layer are $\mu_{a,1}$, $\mu_{s,1}^{\prime }$, and $F_{1}$, respectively. The corresponding properties of the cerebral layer are $\mu_{a,2}$, $\mu_{s,2}^{\prime }$, and $F_{2}$. A point source (S1) is incident on the middle of the cylinder top, and multiple point detectors $\left(D_{1},D_{2},\ldots ,D_{n}\right)$ are positioned at different distances from the source. (b) Using the NIRFASTer package, we generated synthetic data for a $10\times 10\times 10\;{\mathrm{cm}}^{3}$ 2L cube, with an extracerebral layer thickness of ℓ=1.2  cm. (c) We additionally used NIRFASTer to generate synthetic data for a realistic adult head geometry, wherein the scalp and skull were combined to form a homogeneous extracerebral layer, and the CSF, white matter, and gray matter were combined to form a homogeneous cerebral layer. The source and detectors were positioned on the right side of the head, and we used the average skin-to-brain distance under the middle portion of the optical probe [i.e., the average thickness of the 2-cm long gray line in (c)], as ℓ.

We modeled the head as a cylinder with radius a that consists of a homogeneous extracerebral layer of thickness ℓ above an infinitely thick homogeneous cerebral layer [Fig. 1(a)].^23^ The tissue absorption coefficient, reduced scattering coefficient, and blood flow index of the extracerebral layer are $\mu_{a,1}$, $\mu_{s,1}^{\prime }$, and $F_{1}$, respectively. The corresponding properties of the cerebral layer are $\mu_{a,2}$, $\mu_{s,2}^{\prime }$, and $F_{2}$. The index of refraction is assumed to be the same for both tissue layers. Note that we used a cylindrical geometry instead of a slab geometry because the numeric approximation of the cylindrical Green’s function solutions for diffusive light transport is more robust than the laterally infinite 2L solution.^16^ In the model, a point source is incident on the middle of the cylinder top, and multiple detectors are positioned on the cylinder top at different distances from the source. The distance between the source and the i’th detector is $\rho_{i}$. For FD-DOS, the source is radio-frequency intensity modulated light, which produces a diffuse photon density wave in the tissue oscillating at the same frequency.^1^^,^^3^ At each detector position, the wave’s amplitude and phase are measured, i.e., ${\mathrm{AC}}_{\mathrm{meas}}(\rho_{i})$ and $\theta_{\mathrm{meas}}(\rho_{i})$. For DCS, the source is a continuous-wave (CW), long-coherence-length laser. At each detector position, the detected normalized intensity temporal autocorrelation function, $g_{2}^{\left(\mathrm{meas}\right)}(\rho_{i},\tau )={\left\langle I(t)I(t+\tau )/I(t)\right\rangle }^{2}$, is computed at multiple delay times, τ; here I(t) is the detected light intensity at time t, and the angular brackets, ⟨⟩, represent time averages.

To evaluate the 2L fitting algorithm, we used simulated data for 8 FD-DOS source–detector separations ($\rho_{i}=0.8$ to 4.0 cm) and 2 DCS source–detector separations ($\rho_{i}=0.8$ and 2.5 cm) across a wide range of blood flows and optical properties.

### 2.2 Forward Model Simulations

Our first synthetic dataset was generated using the analytical Green’s function solutions of the frequency-domain photon diffusion equation and the correlation diffusion equation for the 2L cylinder geometry (subject to the extrapolated-zero boundary condition). The frequency-domain Green’s function (i.e., $\Phi_{2L}(\rho_{i})={\mathrm{AC}}_{\mathrm{theo},2L}(\rho_{i})\exp(-i\theta_{\mathrm{theo},2L}(\rho_{i}))$) in this geometry was derived elsewhere^16^^,^^23^ and is presented in Appendix A. The corresponding correlation diffusion Green’s function (i.e., $G_{1}^{\left(\mathrm{theo},2L\right)}(\rho_{i},\tau )$) is also presented in Appendix A. It has the same form as the Green’s function solution of the continuous wave photon diffusion equation, with the new feature of the decay constant in the solution depending on τ.^1^

The frequency-domain solution depends on the tissue optical properties (i.e., $\mu_{a,1}$, $\mu_{s,1}^{\prime }$, $\mu_{a,2}$, and $\mu_{s,2}^{\prime }$), the source–detector separation ($\rho_{i}$), the cylinder radius (a), the extracerebral layer thickness (ℓ), the source intensity modulation frequency (f), and the tissue index of refraction (n). All FD-DOS data were generated using f=110  MHz, a=30  cm, and n=1.4. Note that 110 MHz is a commonly used modulation frequency in FD-DOS instrumentation (e.g., Imagent, ISS), and a was sufficiently large such that the Green’s function solution at the detector positions is not affected by the cylindrical border.

We evaluated the frequency-domain Green’s function at eight different $\rho_{i}$ (see Sec. 2.1) across a wide range of optical properties for four evenly spaced ℓ between 1.0 and 1.6 cm (this range approximates the range of thicknesses for adult humans ^24^^–^^26^). Specifically, at each ℓ value, the Green’s function was evaluated for 2030 different combinations of $\mu_{a,1}$, $\mu_{s,1}^{\prime }$, $\mu_{a,2}$, and $\mu_{s,2}^{\prime }$. To mimic the range of properties observed in adult humans for the 700 to 900 nm spectral range,^27^^–^^29^ $\mu_{a,1}$ and $\mu_{a,2}$ were randomly selected between 0.08 and $0.18\;{\mathrm{cm}}^{-1}$, and $\mu_{s,1}^{\prime }$ and $\mu_{s,2}^{\prime }$ were randomly selected between 6 and $15\;{\mathrm{cm}}^{-1}$ subject to the constraint that the fractional difference between $\mu_{s,1}^{\prime }$ and $\mu_{s,2}^{\prime }$ was <20%. Of note, this latter constraint is justified by a recent study that observed considerable variations in overall scattering across the near-infrared spectral range but small scattering differences between skin, skull, and brain tissue.^28^

Random amplitude and phase noise derived from a Gaussian noise model with zero mean was then independently generated for each source–detector separation and each combination of optical properties. For the amplitude, we generated data with a signal-to-noise ratio (SNR) defined as SNR≡μ/σ=100. For the phase, we added noise with a standard deviation equal to 0.1 deg. These amplitude and phase noise levels were chosen based on previously published *in vivo* data in adults.^30^ We assumed that noise was independent of wavelength and source–detector separation. This roughly resembles the case in practice wherein the detected intensities at short source–detector separations are attenuated to approximately the same scale as the intensities at longer source–detector separations (i.e., to reduce the dynamic range of detection across separations). However, it will be interesting to consider more complex noise models in future work.

The correlation diffusion Green’s function solution depends on the same parameters as the frequency-domain solution and additionally depends on the blood flow indices ($F_{1}$ and $F_{2}$), light wavelength (λ), and delay-time (τ). For λ=785  nm, we evaluated the solution at two $\rho_{i}$ (0.8 and 2.5 cm) and 100 different τ (spanning 0.6  μs to 3.7 ms in a multitau scheme^31^). Specifically, for each FD-DOS optical properties combination, the correlation diffusion solution was evaluated for a randomly selected $F_{1}$ and $F_{2}$ combination. To mimic adult humans, $F_{1}$ was selected between $10^{-9}$ and $2\times 10^{-8}\;{\mathrm{cm}}^{2}/s$, and $F_{2}$ was selected between $10^{-9}$ and $10^{-7}\;{\mathrm{cm}}^{2}/s$. The normalized intensity autocorrelation function was then obtained via the Siegert relation,^1^^,^^32^ i.e., $g_{2}^{\left(\mathrm{theo},2L\right)}(\rho_{i},\tau )=1+\beta {\left|G_{1}^{\left(\mathrm{theo},2L\right)}(\rho_{i},\tau )/G_{1}^{\left(\mathrm{theo},2L\right)}(\rho_{i},0)\right|}^{2}$, where β=0.5 was assumed.

Intensity autocorrelation noise, derived for three different integration times (i.e., T=0.1, 1, and 10 s), was independently added to each $g_{2}^{\left(\mathrm{theo},2L\right)}(\rho_{i},\tau )$ evaluation to obtain synthetic DCS data as a function of T, i.e., $g_{2}^{\left(\mathrm{meas},T\right)}(\rho_{i},\tau )$. The autocorrelation noise was derived using a correlation noise model^33^ evaluated with DCS photon count rates of 200 and 40 kHz for the short and long source–detector separations, respectively. Note that 40 kHz is on the high end for the 2.5-cm source–detector separation, but it is still within the range observed in previously published *in vivo* measurements on adults.^34^ We added random Gaussian noise (with zero mean and a standard deviation based on the correlation noise model described above) independently for each delay-time and source–detector separation and independently for each combination of optical properties and flow indices.

### 2.3 NIRFASTer Simulations

We used the open-source finite-element software package NIRFASTer^35^^,^^36^ to generate additional synthetic datasets for the same set of eight FD-DOS ($\rho_{i}=0.8$, 1.2, 1.6, 2.0, 2.8, 3.2, 3.6, and 4.0 cm) and two DCS source–detector separations ($\rho_{i}=0.8$ and 2.5 cm) placed in two different geometries. The first geometry was a $10\times 10\times 10\;{\mathrm{cm}}^{3}$ 2L cube [Fig. 1(b)] with a node size of 0.07 cm, which provided a final mesh containing 482,460 nodes. The extracerebral layer thickness and absorption coefficient were set to ℓ=1.2  cm and $\mu_{a,1}=0.1\;{\mathrm{cm}}^{-1}$, respectively. The reduced scattering coefficients of both layers in the cube were set to the same value, i.e., $\mu_{s}^{\prime }=\mu_{s,1}^{\prime }=\mu_{s,2}^{\prime }=10\;{\mathrm{cm}}^{-1}$, and held constant. NIRFASTer was then used to simulate $\mathrm{AC}(\rho_{i})$ and $\theta (\rho_{i})$ via a finite-element method for 11 evenly spaced cerebral layer absorption coefficients ($\mu_{a,2}$) between 0.08 and $0.18\;{\mathrm{cm}}^{-1}$. Similar to the forward-model simulations, Gaussian noise was added to $\mathrm{AC}(\rho_{i})$ and $\theta (\rho_{i})$ to obtain 20 different pairs of ${\mathrm{AC}}_{\mathrm{meas}}(\rho_{i})$ and $\theta_{\mathrm{meas}}(\rho_{i})$ synthetic data for each value of $\mu_{a,2}$ (amplitude SNR=100; phase σ=0.1  deg).

For each combination of optical properties, NIRFASTer was also used to generate $G_{1}^{\left(\mathrm{theor},2L\right)}(\rho_{i},\tau )$ via a finite-element method for 16 different CBF indices between $10^{-9}$ and $10^{-7}\;{\mathrm{cm}}^{2}/s$ (the extracerebral flow index was held constant at $F_{1}=10^{-8}\;{\mathrm{cm}}^{2}/s$). Then as described in Sec. 2.2, correlation noise was added to $G_{1}^{\left(\mathrm{theor},2L\right)}(\rho_{i},\tau )$ for a given DCS integration time T to obtain a synthetic DCS measurement (i.e., $g_{2}^{\left(\mathrm{meas},T\right)}(\rho_{i},\tau )$) independently for each source–detector separation. For each integration time (T=0.1, 1, and 10 s) and each combination of optical properties, flow indices, and noise additions from FD-DOS, 15 synthetic DCS measurements were generated (in total, we generated 300 autocorrelation curves for each source–detector separation at each combination of optical property, flow, and integration time).

The second geometry was a realistic adult head mesh created using an open-source library (brain2mesh, with a Delaunay sphere radius of 0.11 cm, radius-to-edge ratio of 1.24, and maximum element volume of $4\;{\mathrm{mm}}^{3}$).^37^ The head was segmented into the scalp, skull, cerebral spinal fluid (CSF), white matter, and gray matter, containing ∼1.4 million nodes. We removed the nodes further than 10 cm from the simulated source, reducing the final mesh to 663,470 nodes. For our simulations, the scalp and skull were merged to form one homogeneous tissue type (i.e., the extracerebral layer), whereas the CSF, gray matter, and white matter were merged to form a second homogeneous tissue type (i.e., the cerebral layer). The synthetic data for this geometry were generated with NIRFASTer in the same manner as the cube simulations (including the same combinations of extracerebral and cerebral tissue properties and noise additions). Note that, although the scalp and skull blood flows are quite different under normal conditions, the concatenation of the scalp and skull into one layer is closer to reality for applications wherein a transient high probe pressure can be applied against the scalp to reduce the scalp flow closer to levels in the skull.^12^ Our results are thus most relevant for these conditions.

We did, however, conduct a pilot test of the algorithm under conditions of the scalp flow being higher than the skull flow. This test used simulated data for the same realistic head geometry, except that the scalp and skull tissues were assigned distinct optical properties and flow indices (i.e., a three-layer realistic head geometry). Specifically, we simulated data in which the absorption coefficients for the scalp and skull were $\mu_{\text{scalp}}=0.1\;{\mathrm{cm}}^{-1}$ and $\mu_{\text{skull}}=0.15\;{\mathrm{cm}}^{-1}$,^28^ respectively, and the blood flow indices were equal to $F_{\text{scalp}}=10^{-8}{\;\mathrm{cm}}^{2}/s$ and $F_{\mathrm{skull}}=10^{-9}{\mathrm{cm}}^{2}/s$, respectively. Here we varied the true cerebral absorption coefficient ($\mu_{a,2,\mathrm{act}}$) between 0.08 and $0.16\;{\mathrm{cm}}^{-1}$ (in steps of $0.02\;{\mathrm{cm}}^{-1}$). For each change in cerebral absorption, we also varied the cerebral flow ($F_{2,\mathrm{act}}$) between 4 and $10\times 10^{-8}\;{\mathrm{cm}}^{2}/s$ (in steps of $10^{-8}\;{\mathrm{cm}}^{2}/s$). As with the other simulations, homogeneous scattering was assumed (i.e., $\mu_{s,\text{scalp}}^{\prime }=\mu_{s,\text{skull}}^{\prime }=\mu_{s,2}^{\prime }=10\;{\mathrm{cm}}^{-1}$), and we added random Gaussian noise to generate multiple datasets from each simulation.

### 2.4 Liquid Phantom Experiments

**Fig. 2:**
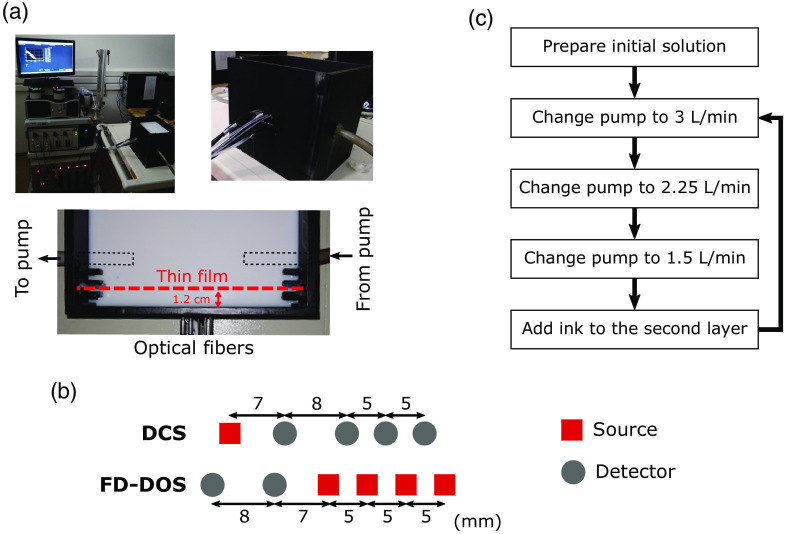
2L phantom setup. (a) We used a hybrid diffuse optical system composed of an FD-DOS and a DCS module. For the phantom experiment, we developed a black acrylic aquarium capable of mimicking a 2L geometry. To separate each layer, we inserted a black frame with a thin plastic film. The second layer of the phantom was connected to a peristaltic pump. (b) The experiment was performed with an optical sensor that allowed for measurements with eight different SDS (ρ=0.7, 1.2, 1.5, 1.7, 2.0, 2.2, 2.5, and 3.0 cm) for FD-DOS and four SDS (ρ=0.8, 1.5, 2.0, and 2.5 cm) for DCS. (c) We varied the second layer’s absorption a total of eight times by adding an ink solution to the second layer. For the 2L phantom experiment, we also varied the flow in the second layer by varying the flow output of the peristaltic pump after each ink addition.

Finally, we performed *in vitro* testing of the algorithm in a 2L liquid phantom. To this end, we developed a custom black acrylic aquarium to mimic a 2L cube [Fig. 2(a)]. We used a removable thin plastic film attached to a black frame to separate the two layers. To attach the optical fibers, we drilled holes in one side of the phantom, such that fibers were in direct contact with the liquid. We glued neutral-density filters in some fiber positions to avoid saturation of the shorter source–detector separations. Finally, to allow flow changes in the second layer, we also attached a peristaltic pump to the aquarium’s lateral sides [Fig. 2(a)].

**Fig. 3:**
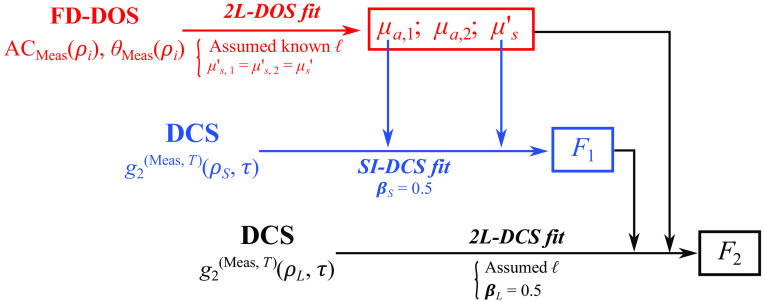
2L fitting scheme. The scheme assumes *a priori* knowledge of the extracerebral layer thickness (ℓ). First, we fit the multidistance FD-DOS amplitude (${\mathrm{AC}}_{\mathrm{meas}}(\rho_{i})$) and phase ($\theta_{\mathrm{meas}}(\rho_{i})$) data to the two-layer (2L) cylindrical frequency-domain Green’s function solution to recover the extracerebral and cerebral layer absorption coefficients ($\mu_{a,1}$, $\mu_{a,2}$), and the reduced scattering coefficient ($\mu_{s}^{\prime }$; the scheme assumes $\mu_{s,1}^{\prime }=\mu_{s,2}^{\prime }=\mu_{s}^{\prime }$). Next, using the recovered $\mu_{a,1}$ and $\mu_{s}^{\prime }$ as inputs, we fit the DCS measurement at the short source–detector separation ($g_{2}^{\left(\mathrm{meas},T\right)}(\rho_{S},\tau )$) to the SI correlation diffusion Green’s function solution to recover the extracerebral flow index ($F_{1}$), assuming knowledge of the β factor from Siegert’s relation ($\beta_{s}=0.5$). Finally, using $\mu_{a,1}$, $\mu_{a,2}$, $\mu_{s}^{\prime }$, and $F_{1}$ as inputs, we fit the DCS measurements at a long source–detector separation ($g_{2}^{\left(\mathrm{meas},T\right)}(\rho_{L},\tau )$) to the 2L cylindrical Green’s function solution to recover the cerebral flow index ($F_{2}$), assuming $\beta_{L}=0.5$.

For our phantom experiments, we used a hybrid diffuse optical system developed at the University of Campinas, which is described elsewhere.^38^^,^^39^ Briefly, the system combines a commercial FD-DOS system (Imagent, ISS) and a homemade DCS system. The FD-DOS system contains 4 detectors and 32 lasers equally split among 4 wavelengths (685, 705, 750, and 830 nm). The DCS system contains a single-laser emitting at 785 nm (CrystaLaser) and 16 single-photon counters (SPCM-AQ4C, Excelitas). We used two detectors and 16 sources from the FD-DOS module for the experimental phantom measurements and one source and twelve detectors from the DCS module (two detectors at 0.7 cm, four at 1.5 cm, three at 2 cm, and four at 2.5 cm). Our phantom [Fig. 3(b)] allowed for FD-DOS measurements with eight different source–detector separations (ρ=0.7, 1.2, 1.5, 1.7, 2.0, 2.2, 2.5, and 3.0 cm), and DCS measurements with four source–detector separations (ρ=0.7, 1.5, 2.0, and 2.5 cm). Prior to each experiment, the FD-DOS measurements were calibrated using three different solid phantoms, as described elsewhere.^39^^,^^40^

Using the setup described above, we performed two separate phantom experiments. In the first experiment, we measured the phantom in a homogeneous geometry (i.e., without the thin plastic film separating the layers). We started with a solution consisting of 4.5 L of distilled water, 200 mL of Liponfundin 20%, and 2 mL of an ink solution. The ink solution was made with 0.5 mL of ink (Nankin, Acrilex) and 25 mL of distilled water. After measuring the initial solution, we increased the homogeneous medium’s absorption coefficient by repeatedly adding 0.5 mL of the ink solution to the phantom; in total, we simulated eight different absorption coefficients. After each ink addition, we waited at least 6 min for the solution to reach equilibrium before moving to the next step. The data from this homogeneous experiment were used to estimate the true optical properties for different volume ratios of the liquid phantom.

In the second experiment, we started with a solution with the same ink and intralipid concentrations used in the start of the homogenous phantom experiment described above. However, before any ink additions, we placed a thin plastic film at 1.2 cm from the phantom wall to simulate a 2L geometry; data collection started after placing the thin film. Here we varied the absorption coefficient of the second layer via multiple additions of 0.5 mL of the ink solution. After each ink addition, we also changed the second layer’s flow by changing the flow output of a peristaltic pump between 1.5 and 3  L/min [Fig. 3(c)]. Similar to the homogeneous phantom experiment, we waited at least 6 min after each absorption and flow change step, and we repeated this process to simulate eight distinct second-layer absorption coefficients.

### 2.5 Two-Layer Fitting Algorithm

The 2L fitting scheme is depicted in Fig. 3. The scheme assumes that homogeneous tissue reduced scattering (i.e., $\mu_{s,1}^{\prime }=\mu_{s,2}^{\prime }=\mu_{s}^{\prime }$; we performed an analysis to justify this assumption, which we discuss below) and that the extracerebral layer thickness is known *a priori*. We first used a nonlinear constrained global optimizer implemented in MATLAB R2020a (fmincon, Mathworks, Natick, Massachusetts, United States) to obtain estimates of $\mu_{a,1}$, $\mu_{a,2}$, and $\mu_{s}^{\prime }$ by fitting multidistance FD-DOS data (i.e., ${\mathrm{AC}}_{\mathrm{meas}}(\rho_{i})$, $\theta_{\mathrm{meas}}(\rho_{i})$) to the 2L analytical frequency-domain Green’s function solution (i.e., ${\mathrm{AC}}_{\mathrm{theo},2L}(\rho_{i})$ and $\theta_{\mathrm{theo},2L}(\rho_{i})$, see Sec. 2.2). Specifically, we used fmincon to find the set of $\mu_{a,1}$, $\mu_{a,2}$, and $\mu_{s}^{\prime }$ parameters that minimize the cost function $\chi_{\mathrm{FD}}=\sqrt{\chi_{\mathrm{AC}}^{2}}+\sqrt{\chi_{FD}^{2}}$, where $\chi_{\mathrm{AC}}$ and $\chi_{\theta }$ are defined as

$$\chi_{\mathrm{AC}}^{2}=\overset{N}{\underset{i\;=\;1}{\sum }}{\left[\log\;\frac{{\mathrm{AC}}_{\mathrm{meas}}(\rho_{i})}{{\mathrm{AC}}_{\mathrm{meas}}(\rho_{1})}-\log\;\frac{{\mathrm{AC}}_{\mathrm{theo},2L}(\rho_{i})}{{\mathrm{AC}}_{\mathrm{theo},2L}(\rho_{1})}\right]}^{2},$$ (1)

and

$$\chi_{\theta }^{2}=\overset{N}{\underset{i=1}{\sum }}{\left[(\theta_{\mathrm{meas}}(\rho_{i})-\theta_{\mathrm{meas}}(\rho_{1}))-(\theta_{\mathrm{theo},2L}(\rho_{i})-\theta_{\mathrm{theo},2L}(\rho_{1}))\right]}^{2}.$$ (2)

Here N=8 is the total number of source–detector distances. The minimization was also subject to the following constraints: $0.005\leq \mu_{a,1}({\mathrm{cm}}^{-1})<0.6$, $0.005\leq \;\mu_{a,2}\;({\mathrm{cm}}^{-1})<0.6$, and $4\leq \mu_{s,i}^{\prime }\;({\mathrm{cm}}^{-1})<20$. These constraints were based on an adult head’s expected ranges of optical properties.^27^^,^^28^ The known extracerebral layer thickness, an index of refraction (n=1.4), and a cylindrical radius (a=30  cm) were used as inputs in the minimization, and the initial guesses used for $\mu_{a,1}$, $\mu_{a,2}$, and $\mu_{s}^{\prime }$ in the minimization were 0.1, 0.1, and $10\;{\mathrm{cm}}^{-1}$, respectively. The normalization of the amplitude and phase data by their values at the shortest source–detector distance in the cost functions removes the need to fit for additional amplitude and phase scaling factors. Additionally, because the amplitude decreases exponentially with increasing the source–detector distance, we used the logarithm of the amplitude to minimize bias to the shorter distances (i.e., such that fitting errors at each distance are weighted approximately evenly in the cost function).

In the next step, we fit the short-separation DCS data (i.e., $g_{2}^{\left(\mathrm{meas},T\right)}\;(\rho_{s},\tau )$, see Secs. 2.2 and 2.3) to the SI correlation diffusion solution to obtain the extracerebral flow index $F_{1}$ given the $\mu_{a,1}$ and $\mu_{s}^{\prime }$ inputs determined from FD-DOS. The short separation (i.e., $\rho_{s}=0.8\;\mathrm{cm}$) was chosen such that the detected light is predominantly confined to the extracerebral layer for the adult head geometry.^10^^,^^41^ The use of a homogeneous SI tissue model for the short-separation data is thus reasonable. We employed the same nonlinear optimizer (fmincon) to find an $F_{1}$ value that minimizes the cost function $\chi_{\mathrm{DCS},\rho_{S}}$, which is defined as

$$\chi_{\mathrm{DCS},\rho_{S}}^{2}={\underset{\tau_{i}}{\sum }(g_{2}^{\left(\mathrm{meas},T\right)}(\rho_{S},\tau_{i})-(1+\beta_{S}{\left|\frac{G_{1}^{\left(\mathrm{theo},\mathrm{SI}\right)}(\rho_{S},\tau_{i})}{G_{1}^{\left(\mathrm{theo},\mathrm{SI}\right)}(\rho_{S},0)}\right|}^{2}))}^{2}.$$ (3)

Here $\tau_{i}$ was summed over values satisfying the limit $g_{2}^{\left(\mathrm{meas},T\right)}(\rho_{s},\tau )\geq 1$; $G_{1}^{\left(\mathrm{theo},\mathrm{SI}\right)}(\rho_{s},\tau )$ is the analytical Green’s function solution to the correlation diffusion equation for the SI homogeneous geometry (see Appendix A); and $\beta_{S}$ is the Siegert relation coefficient for the short separation, assumed to be 0.5. The minimization was constrained within $10^{-11}\leq F_{1}\leq 10^{-5}{\;\mathrm{cm}}^{2}/s$, and the initial guess for $F_{1}$ in the minimization was $10^{-8}\;{\mathrm{cm}}^{2}/s$.

In the third and final step, we fit the long-separation DCS data [i.e., $g_{2}^{\left(\mathrm{meas},T\right)}(\rho_{L},\tau )$, see Secs. 2.2 and 2.3] to the 2L correlation diffusion solution to obtain the cerebral flow index $F_{2}$ given the inputs of $\mu_{a,1}$, $\mu_{a,2}$, $\mu_{s}^{\prime }$, and $F_{1}$ from the first two steps. Additional inputs in the fit were the extracerebral layer thickness, index of refraction, DCS wavelength (λ=785  nm), the cylindrical radius of a=30  cm, and $\beta_{L}=0.5$. We used fmincon to find the $F_{2}$ value that minimizes the cost function $\chi_{\mathrm{DCS},\rho_{L}}$, which is defined as

$$\chi_{\mathrm{DCS},\rho_{L}}^{2}=\underset{\tau_{i}}{\sum }{\left(g_{2}^{\left(\mathrm{meas},T\right)}(\rho_{L},\tau_{i})-(1+\beta_{L}{\left|\frac{G_{1}^{\left(\mathrm{theo},2L\right)}(\rho_{L},\tau_{i})}{G_{1}^{\left(\mathrm{theo},2L\right)}(\rho_{L},0)}\right|}^{2})\right)}^{2}.$$ (4)

Here $\tau_{i}$ was summed over values satisfying the limit $g_{2}^{\left(\mathrm{meas},T\right)}(\rho_{l},\tau )\geq 1$; $G_{1}^{\left(\mathrm{theo},2L\right)}(\rho_{L},\tau )$ is the analytical Green’s function solution to the correlation diffusion equation for the 2L cylindrical geometry (see Appendix A); the minimization was subject to $10^{-11}\leq F_{2}\leq 10^{-5}\;{\mathrm{cm}}^{2}/s$; and the initial guess for $F_{2}$ was $10^{-8}\;{\mathrm{cm}}^{2}/s$.

### 2.6 Data Analysis

#### 2.6.1 Accuracy of the two-layer and homogenous approaches

To compare the results of the 2L scheme with the commonly used homogeneous model, we used the SI homogeneous solution of the diffusion equation to recover $F_{\mathrm{SI}}$, $\mu_{a,\mathrm{SI}}$, and $\mu_{s,\mathrm{SI}}^{\prime }$. For this analysis, we focused on the longer source–detector separations: for FD-DOS, we used ρ=2.8, 3.2, 3.6, and 4.0 cm; for DCS. We used ρ=2.5  cm, and we also assumed $\beta_{L}=0.5$ for Siegert’s relation. In addition, we restricted our analysis to $g_{2}^{\left(\mathrm{meas},T\right)}(\rho_{L},\tau )\geq 1.25$ to increase the sensitivity to cerebral tissue.^10^^,^^42^

We applied the homogeneous SI analysis described above and the scheme described in Sec. 2.4 and Fig. 2 to the four synthetic datasets generated with (1) 2L cylindrical diffusion forward model (Sec. 2.2), (2) NIRFASTer in the 2L cube geometry (Sec. 2.3), (3) NIRFASTer in the 2L realistic adult head geometry (Sec. 2.3), and (4) NIRFASTer in the three-layer realistic adult head geometry (Sec. 2.4). Of note, for the realistic adult head geometry, we used the average skin-to-brain distance under the middle portion of the probe [i.e., ℓ=1.22  cm, see Fig. 1(c)] as the extracerebral thickness.

For the primary analysis, we focused only on the 2L synthetic datasets, but we still report the corresponding results for the pilot three-layer synthetic dataset in Appendix C. The primary analysis also involves only the DCS estimates obtained with an integration time of T=10  s. Defining the absolute percent error as 100×|actual−recovered|/actual, we computed the median absolute percent error (MAPE) and the interquartile range (IQR) of the absolute percent errors of the recovered parameters obtained with the constrained 2L and homogeneous fitting algorithms across all simulations in each synthetic dataset (i.e., to convey the overall accuracy and precision). Paired Wilcoxon sign-rank tests were used to compare the MAPE between the 2L and homogeneous reconstructions of the cerebral tissue absorption coefficient and the CBF index. All statistical tests were two-sided, and p<0.05 was considered to indicate significance.

We also plotted the medians and IQRs of the recovered parameters against the actual values in each synthetic dataset (i.e., $F_{i,\mathrm{act}}$, $\mu_{a,i,\mathrm{act}}$, and $\mu_{s,\mathrm{act}}^{\prime }$). The IQRs represent the robustness of the recovered parameters against noise. They are also a measure of the stability of the recovered flow indices against changing optical properties. We further used linear regression to investigate the agreement between (a) the recovered 2L cerebral tissue absorption coefficient ($\mu_{a,2}$) and the actual cerebral tissue absorption coefficient ($\mu_{a,2,\mathrm{act}}$); (b) the recovered SI tissue absorption coefficient ($\mu_{a,\mathrm{SI}}$) and $\mu_{a,2,\mathrm{act}}$; (c) the recovered 2L cerebral flow index ($F_{2}$) and the actual cerebral flow index ($F_{2,\mathrm{act}}$); and (d) the recovered SI flow index ($F_{\mathrm{SI}}$) and $F_{2,\mathrm{act}}$.

#### 2.6.2 Sensitivity of the FD-DOS two-layer Green’s function to tissue optical properties changes

In a secondary analysis, we sought to justify the homogeneous reduced scattering coefficient assumption ($\mu_{s,1}^{\prime }=\mu_{s,2}^{\prime }=\mu_{s}^{\prime }$) by evaluating the sensitivity of the 2L FD-DOS amplitude and phase (i.e., ${\mathrm{AC}}_{\mathrm{theo},2L}(\rho_{i})$ and $\theta_{\mathrm{theo},2L}(\rho_{i})$) to changes in $\mu_{a,1},\mu_{a,2},\mu_{s,1}^{\prime }$, and $\mu_{s,2}^{\prime }$. If the amplitude and phase values are minimally sensitive to changes in $\mu_{s,2}^{\prime }$, we argue that the extraction of all four optical properties from fitting the FD-DOS data to the 2L model will be inaccurate because of high crosstalk between $\mu_{s,2}^{\prime }$ and the other fitting parameters. Instead, it is better to assume homogenous reduced scattering, especially given the evidence from a recent study that found small scattering differences between skin, skull, and brain.^28^ To assess the sensitivities, we computed the derivatives $\partial \;\log\;{\mathrm{AC}}_{\mathrm{theo},2L}(\rho_{i})/\partial x_{i}$ and $\partial \theta_{\mathrm{theo},2L}(\rho_{i})/\partial x_{i}$, where $x_{i}$ refers to $\mu_{a,1},\mu_{a,2},\mu_{s,1}^{\prime }$, and $\mu_{s,2}^{\prime }$. The derivatives for each parameter were evaluated at source–detector distances between 0.8 and 5 cm for the tissue properties at the midpoints of the ranges used for the forward simulations (Sec. 2.2; $\mu_{a,1}=\mu_{a,2}=0.13\;{\mathrm{cm}}^{-1}$, $\mu_{s,1}^{\prime }=\mu_{s,2}^{\prime }=10.5\;{\mathrm{cm}}^{-1}$, ℓ=1.2  cm).

#### 2.6.3 Errors arising from the assumption of homogeneous tissue reduced scattering

In another secondary analysis of the forward model synthetic dataset, we characterized the effects on the errors of the recovered $F_{2}$ and $\mu_{a,2}$ values when, in contrast to our algorithm’s assumption, the actual tissue reduced scattering is not homogenous (i.e., $\mu_{s,1,\mathrm{act}}^{\prime }\neq \mu_{s,2,\mathrm{act}}^{\prime }$). For the 1.0, 1.2, and 1.6 cm extracerebral thicknesses in the forward-model dataset, we compared the percent errors for $F_{2}$ and $\mu_{a,2}$ against the ratio of the actual cerebral and extracerebral reduced scattering coefficients ($\mu_{s,2,\mathrm{act}}^{\prime }/\mu_{s,1,\mathrm{act}}^{\prime }$). Specifically, we discretized $\mu_{s,2,\mathrm{act}}^{\prime }/\mu_{s,1,\mathrm{act}}^{\prime }$ into 20 evenly spaced bins from 0.8 to 1.2 and plotted the median and IQR of the percent errors (100×(actual−recovered)/actual) across the simulations run within each bin.

#### 2.6.4 Error arising from the assumption of homogeneous tissue absorption

In a third secondary analysis of a subset of the synthetic data generated for the 2L cube and realistic head geometries, we investigated how the assumption of homogeneous tissue absorption affects the recovered $F_{2}$ accuracy (e.g., to evaluate the accuracy of using homogeneous absorption as an additional constraint for reconstructing $F_{2}$). In both geometries, we selected data for which $F_{1,\mathrm{act}}$, $F_{2,\mathrm{act}}$, $\mu_{a,1,\mathrm{act}}$, $\mu_{s,1,\mathrm{act}}^{\prime }$, and $\mu_{s,2,\mathrm{act}}^{\prime }$ were fixed at $10^{-8}{\;\mathrm{cm}}^{2}/s$, $6\times 10^{-8}{\;\mathrm{cm}}^{2}/s$, $0.1{\;\mathrm{cm}}^{-1}$, $10{\;\mathrm{cm}}^{-1}$, and $10\;{\mathrm{cm}}^{-1}$, respectively, whereas $\mu_{a,2,\mathrm{act}}$ varied between 0.08 and $0.18\;{\mathrm{cm}}^{-1}$. We then reapplied the 2L algorithm to recover $F_{2}$ under the additional assumptions that $\mu_{a,1}=\mu_{a,2}=\mu_{a,\mathrm{SI}}$ and $\mu_{s,1}^{\prime }=\mu_{s,2}^{\prime }=\mu_{s,\mathrm{SI}}^{\prime }$ (i.e., assuming heterogeneous flow but homogeneous optical properties). These recovered values, along with the original recovered $F_{2}$ values (i.e., from our primary analysis in which homogeneous absorption was not assumed), were plotted against $\mu_{a,2,\mathrm{act}}$.

#### 2.6.5 Error arising from DCS correlation noise

In a fourth secondary analysis of all three 2L synthetic datasets (forward model, cube, and realistic head), we assessed the influence of DCS correlation noise on the accuracies of the recovered $F_{2}$ and $F_{\mathrm{SI}}$. The MAPE [IQR] of the recovered values was determined for each DCS integration time (i.e., T=0.1, 1, and 10 s). Correlation noise is higher at shorter T. Thus if correlation noise considerably affects the accuracy, then absolute percent errors and IQR in the recovered values will be substantially worse for T=0.1  s and/or 1 s than for T=10  s.

In a related analysis, we estimated the contrast-to-noise ratio (CNR) at each integration time for $F_{2}$ and $F_{\mathrm{SI}}$ in the realistic head geometry for an actual CBF increase of 50%. We computed CNR as $\mathrm{CNR}=\text{median}(\Delta F_{i})/\mathrm{std}(F_{i})$, where $\Delta F_{i}$ is the flow difference recovered (either $F_{2}$ or $F_{\mathrm{SI}})$ for the simulations with $F_{2,\mathrm{act}}=4$ and $6\times 10^{-8}\;{\mathrm{cm}}^{2}/s$. The median was calculated across all different noise simulations with fixed flow changes and across all simulated values for the cerebral absorption. The standard deviation $\left(\mathrm{std}(F_{i})\right)$ was calculated across all simulations at the baseline value (i.e., when $F_{2,\mathrm{act}}=4\times 10^{-8}\;{\mathrm{cm}}^{2}/s$).

#### 2.6.6 Error arising from inaccurate extracerebral thickness

The final secondary analysis estimates the sensitivities of the recovered $F_{2}$ and $\mu_{a,2}$ to the extracerebral layer thickness in the 2L realistic adult head geometry. We applied the 2L fitting algorithm using nine evenly spaced ℓ between 1.0 and 1.4 cm. For each ℓ, the algorithm was applied to the same subset of the synthetic data with $F_{2,\mathrm{act}}>F_{1,\mathrm{act}}$. We focused on this subset to mimic the typical case of CBF greater than extracerebral blood flow.^43^ The MAPE [IQR] of the recovered $F_{2}$ and $\mu_{a,2}$ was determined for each ℓ.

#### 2.6.7 Accuracy of the methodology in a two-layered liquid phantom

To test our methodology in a real-world scenario *in vitro*, we performed measurements on a 2L liquid phantom. We focused our analysis on 2-min averages of the measured $\mathrm{AC}(\rho_{i})$, $\theta (\rho_{i})$, and $g_{2}(\rho_{i},\tau )$ at each step in our phantom experiment, and we focused on $\rho_{i}=0.8$ and 2.5 cm for DCS. To obtain the true optical properties of the liquid mixture (i.e., $\mu_{a,1,\mathrm{act}}$, $\mu_{a,2,\mathrm{act}}$, and $\mu_{s,\mathrm{act}}^{\prime }$), we used a homogeneous SI model to fit data from our first phantom experiment (in a homogeneous geometry); in this case, we focused on the longer source–detector separations (i.e., ρ=1.5, 2, 2.5, 3 cm). Importantly, in our second experiment (2L geometry), we used the same volume ratios of the first experiment for the second layer, and we assumed that the optical properties are reproducible when using the same volume ratios.

After obtaining the expected optical properties, we fitted the data from the 2L phantom experiment using our 2L fitting algorithm to recover the optical properties and flow from each layer, as described previously (Sec. 2.4 and Fig. 3). Similar to our primary analysis, we additionally used an SI model to fit the longer source–detector separations (i.e., ρ=1.5, 2, 2.5, 3 cm for FD-DOS and ρ=2.5  cm for DCS) to recover $F_{SI},\mu_{a,\mathrm{SI}}$, and $\mu_{s,\mathrm{SI}}^{\prime }$. We calculated the MAPE and IQR for the recovery of the optical properties from FD-DOS; MAPE was calculated using the optical properties measured for the homogeneous phantom as the ground truth, as described above. Corresponding ground truth flow indices ($F_{i,\mathrm{act}}$), however, could not be estimated the same way because the peristaltic pump flow output is not easily translated to a DCS flow index. Thus to avoid arbitrary assumptions, we opted to not compute MAPE for $F_{1}$, $F_{2}$, and $F_{\mathrm{SI}}$.

Note that, for the DCS fitting, we estimated the β coefficient from the Siegert’s relation using an SI fit of the autocorrelation curves at each source–detector separation; this fit only used the early delay times (i.e., $g_{2}(\rho_{i},\tau )\geq 1.25$), and we fitted for β only once, before any ink was added to the second layer and with the pump at its highest setting (i.e., 3  L/min).

## 3 Results

### 3.1 Accuracy of the Two-Layer and Homogenous Approaches

**Fig. 4:**
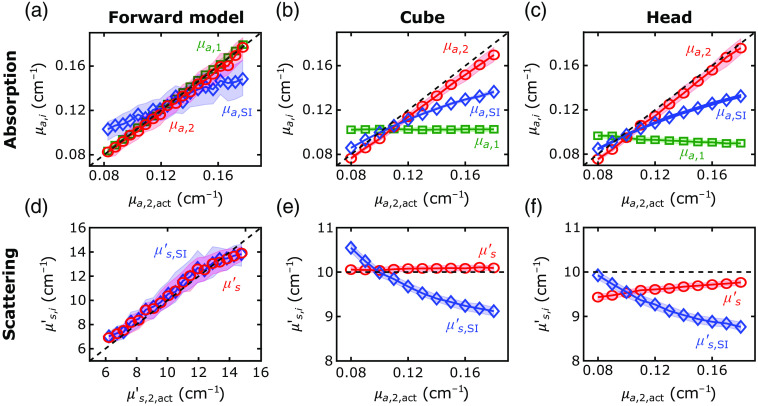
Recovered versus actual tissue optical properties. (a)–(c) The recovered tissue absorption ($\mu_{a,i}$) and tissue reduced scattering coefficient ($\mu_{s,i}^{\prime }\;$) for the extracerebral (green lines) and cerebral (red lines) layers are plotted against the actual values of the second-layer absorption ($\mu_{a,2,\mathrm{act}}$) for the (a), (d) forward-model, (b), (e) cube, and (c), (f) realistic head simulations (circles denote the medians of the recovered values across all simulations run for each actual value; shaded areas represent the IQR). The corresponding recovered tissue absorption ($\mu_{a,\mathrm{SI}}$) and reduced scattering coefficients from the SI model are also plotted against the actual cerebral absorption values (blue diamonds). Dashed lines represent the actual relationships between each parameter and the cerebral absorption coefficient.

The first step of our 2L fitting algorithm was the recovery of the optical properties of each layer from the FD-DOS measures of ${\mathrm{AC}}_{\mathrm{meas}}$ and $\theta_{\mathrm{meas}}$. With our algorithm, we recovered the tissue absorption and reduced scattering coefficients with excellent agreement between the recovered and actual values for the forward-model, 2L cube, and 2L realistic head simulations (see Fig. 4 and Table 1; exemplar ${\mathrm{AC}}_{\mathrm{meas}}$ and $\theta_{\mathrm{meas}}$ fits shown in Appendix B). In these geometries, median errors were <8%. The best-fit linear regression lines for the comparison of $\mu_{a,2}$ and $\mu_{a,2,\mathrm{act}}$ approached the unity line. However, the agreement for the SI analysis was not as good; the slope of the linear best-fit line between $\mu_{a,\mathrm{SI}}$ and $\mu_{a,2,\mathrm{act}}$ (i.e., 0.5) deviated from the unity line.

**Table 1:** MAPE of the optical properties ($\mu_{a,1},\mu_{a,2}$, and $\mu_{s}^{\prime }$) and flow indices ($F_{1}$ and $F_{2}$) recovered with the 2L approach and with the SI approach ($\mu_{a,\mathrm{SI}}$, $\mu_{s,\mathrm{SI}}^{\prime }$, and $F_{\mathrm{SI}}$) for all datasets generated. The linear best-fit relations between the recovered and actual values for the second layer are also reported.

			MAPE [IQR] (%)	Linear regression
Forward-model	Absorption	$\mu_{a,1}$	2.4 [1.1, 4.5]	—
		$\mu_{a,2}$	7 [3, 12]	$1.011\mu_{a,2,\mathrm{act}}-0.002$
		$\mu_{a,\mathrm{SI}}$	11 [5, 19]	$0.51\mu_{a,2,\mathrm{act}}+0.06$
	Scattering	$\mu_{s}^{\prime }$	8 [4, 13]	—
		$\mu_{s,\mathrm{SI}}^{\prime }$	9 [5, 16]	—
	Flow	$F_{1}$	2.4 [1.0, 4.1]	—
		$F_{2}$	7 [3, 13]	$0.98F_{2,\mathrm{act}}+0.05$
		$F_{SI}$	79 [65, 88]	$0.04F_{2,\mathrm{act}}+0.95$
2L cube	Absorption	$\mu_{a,1}$	2.7 [1.4, 4.3]	—
		$\mu_{a,2}$	5.0 [3.0, 7.9]	$0.941\mu_{a,2,\mathrm{act}}+0.001$
		$\mu_{a,\mathrm{SI}}$	10 [5, 19] %	$0.50\mu_{a,2,\mathrm{act}}+0.05$
	Scattering	$\mu_{s}^{\prime }$	0.8 [0.3, 1.4]	—
		$\mu_{s,\mathrm{SI}}^{\prime }$	5.3 [2.8, 7.3]	—
	Flow	$F_{1}$	1.7 [0.8, 2.8]	—
		$F_{2}$	6 [3, 13]	$1.02F_{2,\mathrm{act}}+0.08$
		$F_{\mathrm{SI}}$	69 [35, 80]	$0.08F_{2,\mathrm{act}}+1.03$
Realistic head (2L)	Absorption	$\;\mu_{a,1}$	7.7 [4.7, 9.8]	—
		$\mu_{a,2}$	4.6 [2.4, 7.2]	$1.020\mu_{a,2,\mathrm{act}}-0.007$
		$\mu_{a,\mathrm{SI}}$	12 [6, 21]	$0.47\mu_{a,2,\mathrm{act}}+0.05$
	Scattering	$\mu_{s}^{\prime }$	3.6 [2.8, 4.8]	—
		$\mu_{s,\mathrm{SI}}^{\prime }$	9 [5, 11]	—
	Flow	$F_{1}$	13 [11, 16]	—
		$F_{2}$	34 [30, 42]	$0.70F_{2,\mathrm{act}}-0.09$
		$F_{\mathrm{SI}}$	69 [33, 80]	$0.06F_{2,\mathrm{act}}+1.14$
Liquid phantom (2L)	Absorption	$\;\mu_{a,1}$	10 [9, 11]	—
		$\mu_{a,2}$	8 [5, 12]	$1.24\mu_{a,2,\mathrm{act}}-0.02$
		$\mu_{a,\mathrm{SI}}$	19 [9, 27]	$0.39\mu_{a,2,\mathrm{act}}-0.04$
	Scattering	$\mu_{s}^{\prime }$	10.2 [10.1, 10.4]	—
		$\mu_{s,\mathrm{SI}}^{\prime }$	14 [11, 16]	—

**Fig. 5:**
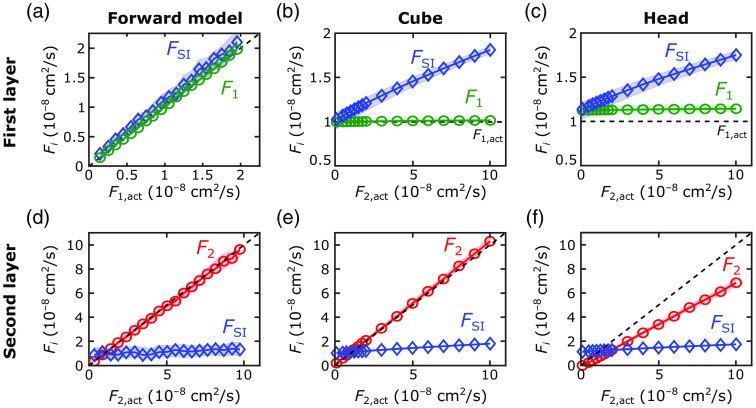
Recovered versus actual flow indices. The (a)–(c) recovered extracerebral flow ($F_{1}$, green lines) and (d)–(f) cerebral flow ($F_{2}$, red lines) indices are plotted against the actual values of the second-layer flow ($F_{2,\mathrm{act}}$) for the [(b), (e)] cube and [(c), (f)] realistic head simulations. For the forward-model simulations [(a), (d)], we plot the extracerebral and CBF values against their actual values. The corresponding recovered flow index ($F_{\mathrm{SI}}$) from the SI model are also plotted against the actual values (blue diamonds). Dashed lines represent the actual relationships between each parameter and the x axis. In all cases, circles denote the medians of the recovered values across all simulations (with varying noise, flow indices, and varying absorption), and shaded areas represent the IQR.

For assessing the reconstructed $F_{1}$ and $F_{2}$ accuracy, we used the simulated datasets with a DCS integration time of T=10  s. For the forward-model and 2L cube simulations, we were able to accurately recover $F_{1}$ with median errors below 3% [Table 1, Figs. 5(a) and 5(b); the exemplar intensity autocorrelation function fits are shown in Appendix B]. For the 2L realistic head simulations, our method of using an SI model to recover $F_{1}$ from a short DCS source–detector separation was modestly less accurate, with errors around 13% [Table 1 and Fig. 5(c)].

We observed excellent agreement between the recovered and actual $F_{2}$ for the forward-model and 2L cube simulations [Table 1 and Figs. 5(d) and 5I]. For both datasets, the errors were <10% on average, and the best-fit linear regression lines approached the unity line (Table 1). In the 2L realistic head simulations, however, the recovered $F_{2}$ systematically underestimated the true value by a median error of 34% [Table 1 and Fig. 5(f)]. The small IQRs of the recovered flow values demonstrate robustness against noise and optical absorption changes.

When neglecting the extracerebral layer using an SI model to estimate $F_{2,\mathrm{act}}$, the systematic errors (i.e., MAPE>69%) were larger than the errors recovered with our 2L approach in all simulated datasets (p<0.001). The SI homogeneous model recovered the correct directional trends for the 2L cube and realistic head simulations, where the first-layer flow was held constant. However, for the forward-model simulations, the recovered $F_{\mathrm{SI}}$ values were highly sensitive to variations in first layer flow [$F_{1,\mathrm{act}}$, Fig. 5(a)]. Note that the small IQRs for $F_{2}$ across variations in extracerebral blood flow indicate minimal cross talk between extracerebral and CBF [Fig. 5(d)].

Finally, for conditions wherein scalp and skull blood flow are substantially different (i.e., the three-layer realistic head geometry), the recovered errors with the constrained 2L algorithm were substantially larger than those for the 2L head simulations, but they were still smaller than the errors in the SI estimates (see Appendix C).

### 3.2 Sensitivity of the FD-DOS Two-Layer Green’s Function to Tissue Optical Property Changes

**Fig. 6:**
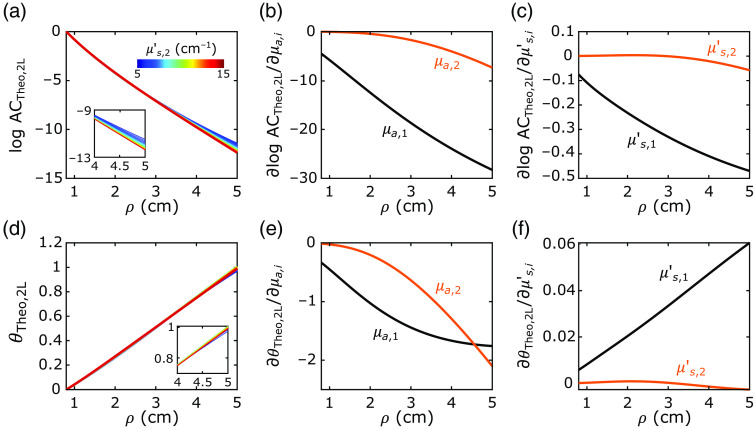
Sensitivity of the 2L FD-DOS Green’s function solution to changes in tissue optical properties. The (a) amplitude logarithm ($\log\;{\mathrm{AC}}_{\mathrm{theo},2L}$) and (d) phase $\left(\theta_{\mathrm{theo},2L}\right)$ of the cylindrical 2L FD-DOS Green’s function solution are plotted against source–detector distance (ρ) for a wide range of cerebral tissue reduced scattering coefficients ($\mu_{s,2}^{\prime }$) between 5 (blue) and $15\;{\mathrm{cm}}^{-1}$ (red). For each $\mu_{s,2}^{\prime }$ evaluation, the extracerebral and cerebral tissue absorption coefficients ($\mu_{a,1}$ and $\mu_{a,2}$) were both fixed at $0.13\;{\mathrm{cm}}^{-1}$, the extracerebral tissue reduced scattering coefficient ($\mu_{s,1}^{\prime }$) was fixed at $10.5\;{\mathrm{cm}}^{-1}$, and the extracerebral layer thickness was fixed at 1.2 cm. The sensitivities of the [(b), (c)] amplitude logarithm [$\partial \;\log\;{\mathrm{AC}}_{\mathrm{theo},2L}/\partial x_{i}$] and [(e), (f)] phase [$\partial \theta_{\mathrm{theo},2L}/\partial x_{i}$] to tissue optical property changes are also plotted against ρ; $x_{i}$ refers to $\mu_{a,1}$, $\mu_{s,1}^{\prime }$, $\mu_{a,2}$, and $\mu_{s,2}^{\prime }$. All derivatives were evaluated at the same optical properties used for (a) and (d) with $\mu_{s,2}^{\prime }$ fixed at $10.5\;{\mathrm{cm}}^{-1}$.

We found that the 2L cylindrical FD-DOS Green’s function solution is minimally sensitive to changes in $\mu_{s,2}^{\prime }$ for source–detector distances (ρ) up to 5 cm (Fig. 6). Variations in the solutions for FD-DOS amplitude (${\mathrm{AC}}_{\mathrm{theo},2L}(\rho )$) and phase ($\theta_{\mathrm{theo},2L}(\rho )$) by variations in $\mu_{s,2}^{\prime }$ from 5 to $15\;{\mathrm{cm}}^{-1}$ are smaller or on the same order of the expected noise [Figs. 4(a) and 4(d)]. Here we fixed the other tissue parameters at ℓ=1.2  cm, $\mu_{a,1}=0.13\;{\mathrm{cm}}^{-1}$, $\mu_{a,2}=0.13\;{\mathrm{cm}}^{-1}$, and $\mu_{s,1}^{\prime }=10.5\;{\mathrm{cm}}^{-1}$. The sensitivities of the FD-DOS amplitude and phase to extracerebral and cerebral layer optical properties are also plotted versus source–detector distance in Fig. 6. The sensitivities are defined by the evaluation of the derivatives $\partial \;\log\;{\mathrm{AC}}_{\mathrm{theo},2L}(\rho_{i})/\partial x_{i}$ and $\partial \theta_{\mathrm{theo},2L}(\rho_{i})/\partial x_{i}$ at $\mu_{a,1}=\mu_{a,2}=0.13\;{\mathrm{cm}}^{-1}$, $\mu_{s,1}^{\prime }=\mu_{s,2}^{\prime }=10.5\;{\mathrm{cm}}^{-1}$, and ℓ=1.2  cm ($x_{i}$ denotes $\mu_{a,1},\mu_{a,2},\mu_{s,1}^{\prime }$, or $\mu_{s,2}^{\prime }$). Note that the sensitivities to $\mu_{s,2}^{\prime }$ are much lower than those for the other optical properties. For example, at ρ=4  cm, the sensitivities of the FD-DOS amplitude and phase to $\mu_{s,2}^{\prime }$ are 5% and −3% of the corresponding sensitivities to $\mu_{s,1}^{\prime }$ and <0.5% of the sensitivities to $\mu_{a,1}$ and $\mu_{a,2}$. Given its minimal sensitivity to the FD-DOS measurements, $\mu_{s,2}^{\prime }$ is not a good fitting parameter. These results justify the need to assume homogeneous tissue reduced scattering.

### 3.3 Error Arising from the Assumption of Homogeneous Tissue Reduced Scattering

**Fig. 7:**
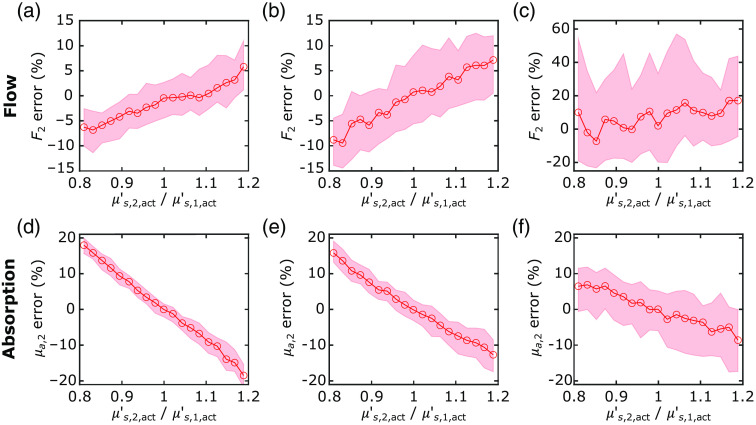
Errors arising from inhomogeneous tissue reduced scattering. Median (circles) and IQRs (shaded area) of the percent errors in the 2L recovered (a)–(c) CBF and (d)–(f) cerebral absorption coefficient plotted against the cerebral to extracerebral ratio of the actual tissue reduced scattering coefficients (the median and IQR are across all simulations ran for each ratio). These plots were generated from the forward-model simulations of three extracerebral thicknesses [(a), (d) ℓ=1.0  cm; (b), (e) ℓ=1.2  cm; and (c), (f) ℓ=1.6  cm].

Although the assumption of homogeneous tissue reduced scattering is justified for 2L fitting, inhomogeneous tissue reduced scattering remains a source of error in the recovery of $F_{2}$ and $\mu_{a,2}$. As a pilot characterization of this issue, we visualized the median percent error of the recovered $F_{2}$ and $\mu_{a,2}$ values as a function of the $\mu_{s,2,\mathrm{act}}^{\prime }/\mu_{s,1,\mathrm{act}}^{\prime }$ ratio in the forward-model simulation dataset. Specifically, the median and IQR of the percent errors for the simulations run at each $\mu_{s,2,\mathrm{act}}^{\prime }/\mu_{s,1,\mathrm{act}}^{\prime }$ ratio are plotted against $\mu_{s,2,\mathrm{act}}^{\prime }/\mu_{s,1,\mathrm{act}}^{\prime }$ for different extracerebral layer thicknesses (Fig. 7). The magnitudes of the errors from inhomogeneous scattering in the recovered $F_{2}$ are smaller than those in the recovered $\mu_{a,2}$. For example, at $\mu_{s,2,\mathrm{act}}^{\prime }/\mu_{s,1,\mathrm{act}}^{\prime }=0.8$ and ℓ=1.2  cm, the median percent errors in the recovered $\mu_{a,2}$ and $F_{2}$ are 15.8% and −8.8%, respectively. Note also that, as ℓ increases, the percent errors are more variable across all $\mu_{s,2,\mathrm{act}}^{\prime }/\mu_{s,1,\mathrm{act}}^{\prime }$ ratios (i.e., the IQR of the errors is larger, see Fig. 7). This larger variability arises because of lower brain sensitivities (fitting of parameters with lower brain sensitivities are more prone to crosstalk caused by measurement noise). Additionally, as the extracerebral thickness increases, the sensitivity of the recovered $\mu_{a,2}$ error to $\mu_{s,2,\mathrm{act}}^{\prime }/\mu_{s,1,\mathrm{act}}^{\prime }$ decreases.

### 3.4 Error Arising from the Assumption of Homogeneous Tissue Absorption

**Fig. 8:**
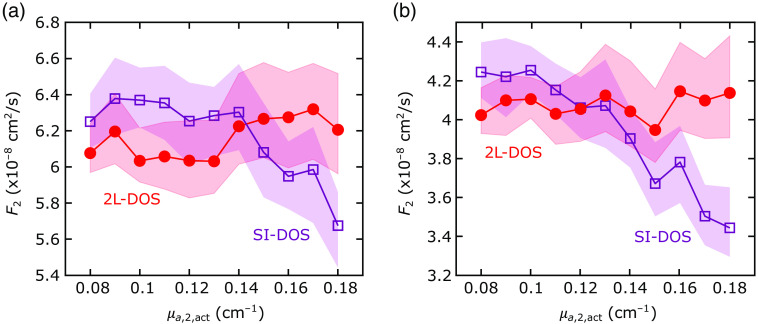
Errors in the recovery of CBF if homogeneous tissue absorption is assumed. The 2L recovered CBF ($F_{2}$) for the (a) cube and (b) 2L realistic head simulations plotted against the actual cerebral tissue absorption coefficient ($\mu_{a,2,\mathrm{act}}$; all other actual tissue flow and optical property parameters were held fixed [see Sec. 2.5)]. $F_{2}$ was recovered using: (1) homogeneous absorption and scattering derived from the SI model (purple squares) and (2) the primary 2L fitting scheme depicted in Fig. 2 (red circles). The purple square and red circles denote the medians across all simulations ran for each $\mu_{a,2,\mathrm{act}}$, and the shaded areas denote the IQRs.

To test the effects of assuming homogeneous tissue absorption as an additional constraint in the recovery of flow indices, we reanalyzed our NIRFASTer simulations using homogeneous optical properties (i.e., using an SI model for FD-DOS, in which we assume $\mu_{a,1}=\mu_{a,2}=\mu_{a,\mathrm{SI}}$ and $\mu_{s,1}^{\prime }=\mu_{s,2}^{\prime }=\mu_{s,\mathrm{SI},}^{\prime }$), but a 2L model for DCS (to separately recover $F_{1}$ and $F_{2}$). Here we focused on cases in which $F_{1,\mathrm{act}}$, $F_{2,\mathrm{act}}$, $\mu_{a,1,\mathrm{act}}$, $\mu_{s,1,\mathrm{act}}^{\prime }$, and $\mu_{s,2,\mathrm{act}}^{\prime }$ were fixed at $10^{-8}\;{\mathrm{cm}}^{2}/s$, $6\times 10^{-8}{\;\mathrm{cm}}^{2}/s$, $0.1\;{\mathrm{cm}}^{-1}$, $10\;{\mathrm{cm}}^{-1}$, and $10{\;\mathrm{cm}}^{-1}$, respectively, whereas $\mu_{a,2,\mathrm{act}}$ varied between 0.08 and $0.18\;{\mathrm{cm}}^{-1}$. When homogeneous tissue absorption was assumed, the actual changes in $\mu_{a,2}$ translated to erroneous changes in $F_{2}$ in both geometries (Fig. 8).

### 3.5 Error Arising from DCS Correlation Noise

**Fig. 9:**
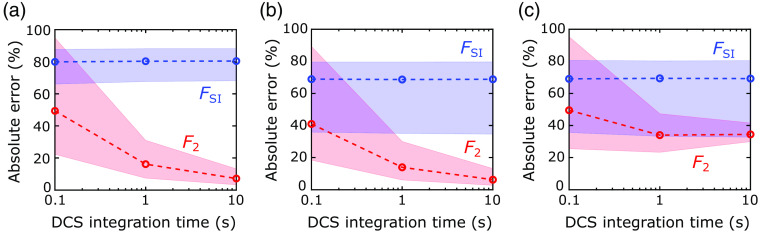
Correlation noise effects on the recovered CBF accuracy. The MAPE (circles) and the IQR of the absolute percent errors (dashed lines) in the SI ($F_{\mathrm{SI}}$) and 2L ($F_{2}$) CBF reconstructions are plotted against DCS integration time (T). The median and IQR were computed across all (a) forward-model simulations, (b) 2L cube simulations, and (c) 2L realistic head simulations ran for each T.

For our fourth secondary analysis, we examined the influence of DCS correlation noise on the accuracy of CBF measurements recovered with the 2L scheme ($F_{2}$) and with the SI scheme ($F_{\mathrm{SI}}$). DCS correlation noise increases with decreasing DCS integration time (T). Correlation noise did not substantially influence the accuracy of the recovered $F_{\mathrm{SI}}$ values in any of the 2L geometries (Fig. 9). The MAPE and IQR of the absolute percent errors were comparable for all three T. For the 2L scheme, however, there was a strong effect (Fig. 9). The MAPE and IQR of the absolute percent errors in $F_{2}$ are noticeably larger at T=0.1  s than at T=10  s for all three geometries. As expected, the 2L model reconstruction is thus more susceptible to correlation noise than the SI model. Note, however, that the reconstruction errors at T=1  s are only minimally to modestly larger than those at T=10  s, depending on the geometry.

Finally, the CNR estimated from the realistic head simulations (see Sec. 2.6.5) for $F_{2}$ at T=0.1, 1 and 10 s were 0.7, 2.5, and 6.0, respectively, and the corresponding CNR for $F_{\mathrm{SI}}$ were 0.7, 1.3, and 1.4, respectively. Note that, although these SI CNR levels were obtained from fitting the upper half of the autocorrelation curve ($g_{2}\geq 1.25$), the SI CNR levels were the same if the entire $g_{2}$ curve was fit for instead ($g_{2}>1$).

### 3.6 Error Arising from Inaccurate Extracerebral Thickness

**Fig. 10:**
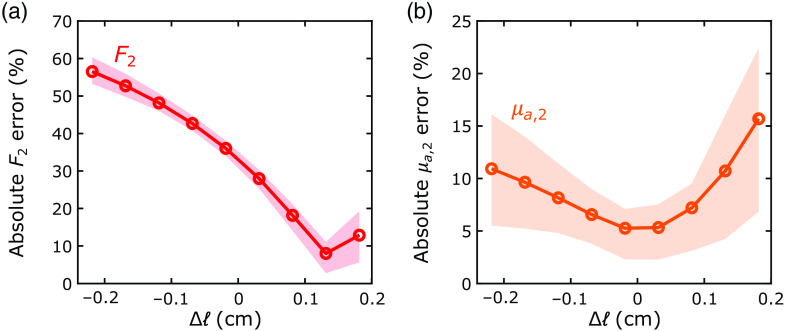
Reconstruction errors in CBF and tissue absorption arising from inaccurate extracerebral layer thickness (ℓ). Errors in the recovered (a) CBF ($F_{2}$) and (b) cerebral tissue absorption coefficient ($\mu_{a,2}$) plotted against errors in the extracerebral layer thickness used for the 2L fits of the realistic head synthetic data. Δℓ is the difference between the extracerebral layer thickness used in the fits and the actual extracerebral layer thickness (1.22 cm). The circles and dashed lines denote the median and IQR of the absolute percent errors across all simulations with actual CBF larger than actual extracerebral blood flow.

We also used the realistic head simulations to investigate the influence of errors in the extracerebral layer thickness on the recovery of $F_{2}$ and $\mu_{a,2}$. The influence was considerable for the $F_{2}$ recovery but more modest for the $\mu_{a,2}$ recovery (Fig. 10). For example, ±0.2 cm errors in ℓ resulted in median errors of up to 15% in $\mu_{a,2}$ and up to 60% in $F_{2}$. Surprisingly, the minimum error for the recovery of $F_{2}$ occurred when the extracerebral layer thickness was overestimated by Δℓ≈0.1  cm.

### 3.7 Accuracy of This Methodology in a Two-Layer Liquid Phatom

**Fig. 11:**
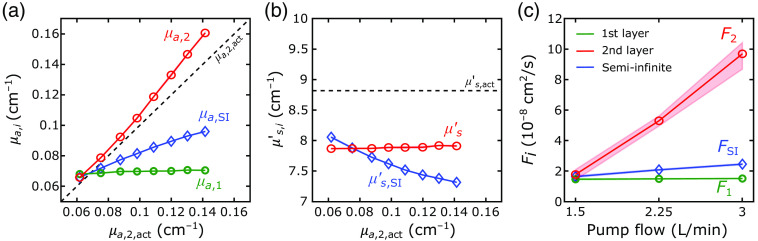
Recovered optical properties and flow indices for the 2L phantom experiment. The 2L and SI (a) recovered absorption coefficients and (b) reduced scattering coefficients are plotted against the actual second-layer values (the actual second-layer values were estimated based on a homogeneous phantom measurement that contained the same volume ratio of ink and intralipid). (c) The 2L and SI recovered flow indices are plotted against the second-layer pump flow used in the experiment. The green lines denote the recovered absorption coefficient ($\mu_{a,1}$) and flow index ($F_{1}$) for the first layer from the 2L fitting algorithm. The red lines denote the recovered absorption coefficient ($\mu_{a,2}$) and flow index ($F_{2}$) for the second layer, as well as the recovered homogenous reduced scattering coefficient ($\mu_{s}^{\prime }$), from the 2L fitting algorithm. Blue lines denote the values recovered using the SI model (i.e., $\mu_{a,\mathrm{SI}}$, $\mu_{s,\mathrm{SI}}^{\prime }$, and $F_{\mathrm{SI}}$). Each point denotes the median of the recovered values across all measurements [i.e., across either varying pump flows for (a) and (b) or varying second-layer absorption for (c); see text]; shaded areas represent the IQR. The IQRs for (a) and (b) were smaller than 0.002 and $0.03\;{\mathrm{cm}}^{-1}$, respectively, and thus are not apparent.

Figure 11 and Table 1 show the comparison of the absolute values recovered with the proposed 2L fitting algorithm and the expected values from the phantom experiment at the 705-nm wavelength. Using our 2L fitting algorithm, we obtained MAPE [IQR] of 10 [9, 11]% for $\mu_{a,1}$, 8 [5, 12]% for $\mu_{a,2}$, and 10.2 [10.1, 10.4]% for $\mu_{s}^{\prime }$. Similar to our simulations, the standard SI homogeneous approximation recovered the optical properties with larger errors (MAPE [IQR] equal to 19 [9, 27]% for $\mu_{a,\mathrm{SI}}$ and 14 [11, 16]% for $\mu_{s,\mathrm{SI}}^{\prime }$). Note that the accuracies for the 685-nm wavelength were very similar to these results (data not shown); the data at the 750 and 830 nm wavelengths were unusable due to calibration and SNR issues.

As expected, the flow indices recovered for the first layer were relatively independent of the second layer’s changes in absorption and flow [Fig. 11(c)]. Additionally, flow indices for the second layer were not entangled with the second layer’s absorption changes [as seen by the small IQR in Fig. 11(c)]. When using an SI homogeneous model to estimate changes in the second layer, we were able to recover the correct trend in the flow changes. However, the absolute values recovered using the SI approximation were significantly lower than those retrieved with our 2L approach. Of note, for the DCS measurements, we recovered β=0.45 for the short SDS (0.7 cm) and 0.49 for the long SDS (2.5 cm).

## 4 Discussion

Using multilayer tissue models is an effective strategy to separate cerebral signals from extracerebral artifacts. Their implementation, however, is often confounded by noise-induced crosstalk in the fitting parameters. To mitigate crosstalk between each parameter, here we used a constrained 2L model in which, instead of fitting for all unknowns simultaneously, the algorithm fits the multidistance FD-DOS and DCS data in sequential steps (Fig. 2). Other constraints are *a priori* knowledge of the extracerebral layer thickness and homogeneous tissue reduced scattering coefficient. We used hybrid FD-DOS and DCS simulations with noise to characterize the algorithm’s accuracy and stability. The simulations were carried out in slab and realistic head geometries and featured typical source–detector distances for cerebral hemodynamic monitoring with DCS (0.8 and 2.5 cm distances) and FD-DOS (0.8 to 4 cm). We found that our constrained 2L algorithm recovered CBF and tissue absorption with higher accuracy than the conventional SI approach. The small IQRs of the parameters recovered across multiple distinct simulations also demonstrate robustness to noise.

The homogeneous reduced scattering assumption is justified by the minimal sensitivity of the FD-DOS signals to changes in cerebral tissue reduced scattering (Fig. 6). Note that this minimal sensitivity was also previously reported for time-domain DOS.^44^ Fitting for a parameter when a signal is minimally sensitive to it leads to increased recovery errors due to increased numerical instability—which might explain the low cerebral reduced scattering coefficients (e.g., $2\;{\mathrm{cm}}^{-1}$ at 830 nm) reported in previous studies that employed multilayer models to analyze DOS data.^16^^,^^29^ One mitigating strategy is to assume the same cerebral tissue scattering coefficient for every subject based on literature values (e.g., from *ex vivo* measurements). Instead, we opted to fit for a homogeneous reduced scattering coefficient based on a prior study that observed similar skin, skull, and brain tissue reduced scattering coefficients.^28^ Note that the minimal sensitivity of FD-DOS signals to changes in cerebral tissue reduced scattering reported herein is valid for adult geometries sampled with source–detector separations ≤5  cm. For applications wherein thinner extracerebral layers are expected (e.g., in children) or larger source–detector separations are used, alternative methodologies that separately recover the reduced scattering coefficient from the first and second layers should be investigated.

A confound of the homogeneous scattering assumption is the observed negative correlation between the error in recovered cerebral absorption and the ratio between the cerebral and extracerebral reduced scattering coefficients (Fig. 7). These errors, however, are still considerably lower than those obtained with the SI approach, and if the errors in the recovered cerebral absorption coefficients are comparable across multiple wavelengths, the cerebral tissue oxygen saturation (${\mathrm{StO}}_{2}$) will still be accurately estimated with multispectral FD-DOS (the errors cancel in the computation of ${\mathrm{StO}}_{2}$).^45^ Interestingly, the recovery of CBF was less affected by the crosstalk between cerebral absorption and the cerebral and extracerebral scattering ratio (Fig. 7). The reduced scattering and absorption coefficients influence the DCS signal in opposite directions,^19^^,^^46^ which partially offset the crosstalk observed when estimating cerebral absorption with our 2L approach.

Surprisingly, the minimum error for the recovery of $F_{2}$ in the realistic head geometry occurred when the extracerebral layer thickness was Δℓ≈0.1  cm higher than our estimation of the “actual” thickness (Fig. 10). This suggests that our method of estimating the actual extracerebral layer thickness was suboptimal. In the realistic head geometry simulated, the skin-to-brain distance varied between 1.09 and 1.33 cm across the length of the optical probe. Recall from Fig. 1 that the estimated actual thickness of ℓ=1.22  cm for the 2L fitting algorithm was obtained by averaging the skin-to-brain distance across the 2-cm-long middle portion of the optical probe. However, if we average the thickness across the 1-cm-long middle portion of the DCS source–detector separation instead, the resulting extracerebral thickness is larger, i.e., ℓ=1.30  cm. Note that this larger thickness is equivalent to the thickness that minimizes the error in the recovery of $F_{2}$ in Fig. 10. These findings, as well as prior studies,^20^^,^^24^ show the importance of the method used for estimating the extracerebral layer thicknesses in multilayer tissue models. Future work is needed to test and optimize estimation methods such as the recently proposed pressure modulation paradigm (which derives an effective layer thickness that differs from direct MRI anatomical measurements)^12^ and the direct fitting of the extracerebral layer thickness.^21^

We also note from the realistic head simulations that the DCS measurement of CBF was more sensitive to errors in the assumed extracerebral layer thickness than the FD-DOS measurement of cerebral tissue absorption (Fig. 10). The higher sensitivity of DCS to extracerebral layer thickness errors is likely explained by the limited DCS brain sensitivity at the 2.5-cm source–detector distance.^24^ Indeed, in a recent simulation study, the DCS measurement was less sensitive to errors in extracerebral layer thickness at a source–detector distance of 3 cm.^47^ Although achieving an adequate SNR at source–detector distances beyond 2.5 cm is challenging, recent studies have demonstrated promising new strategies to boost brain sensitivity.^48^^–^^61^

Previous studies that used a 2L approach for DCS analysis assumed homogeneous or fixed optical properties from the literature.^12^^,^^15^^,^^17^^,^^20^^,^^21^^,^^26^ This approach has the advantage of simplicity. However, a significant disadvantage is the presence of crosstalk between actual cerebral absorption changes and recovered CBF changes, as shown in this work (Fig. 8). When the SI model was used to recover homogeneous absorption and scattering, we found that the recovered CBF with the 2L model varied with changes in cerebral layer absorption, even though actual CBF was constant. Thus the assumption of an optically homogenous medium can lead to wrongly interpreting cerebral oxygenation changes (i.e., changes in $\mu_{a,2}$) as changes in blood flow. For this reason, we recommend using FD-DOS in combination with DCS to separate both cerebral and extracerebral blood flow and tissue optical properties.

A well-known tradeoff of the DCS technique is between high temporal sampling of blood flow and high correlation noise.^33^ Recent work has shown the promise of using a SI model to recover the fast DCS measurements of pulsatile blood flow during the cardiac cycle to assess intracranial pressure.^62^^–^^66^ The use of layered head models can improve accuracy at the cost of higher instability from correlation noise (Fig. 9). The IQR of the absolute percent errors in recovered CBF across all DCS simulations with an integration time of T=0.1  s (or 10 Hz sampling rate) was relatively large for both the SI and 2L approaches (Fig. 9). Thus longer time windows to remove noise via the use of Fourier filtering or averaging across many heart beats^64^ are needed to use 2L algorithms for fast DCS measurements. However, we observed comparable accuracy in blood flow recovery with the 2L model between integration times of T=1  s and T=10  s (Fig. 9).

One concern for the use of multilayer models is the sensitivity of the recovered fitting parameters to the initial guesses for these parameters in the fitting. To evaluate this, we reanalyzed our 2L phantom experiment and a subset of our realistic head simulations using 20 different random initial guesses for each fit (we used the MultiStart function implemented in MATLAB 2020a to this end). Specifically, we reanalyzed the data from all cerebral flow and absorption changes for five of the different noise additions for FD-DOS and DCS. With this approach, the recovered optical properties and flow indices differed by $∼10^{-5}\%$ when compared with our approach of using a fixed initial guess. This reanalysis suggests that our algorithm is numerically stable (i.e., independent of the initial guess used in the fitting procedure).

Beyond computer simulations, we obtained promising *in vitro* results with our algorithm in a 2L liquid phantom. Specifically, we were able to accurately recover the first- and second-layer optical properties with errors ∼10%, which is within the range of expected errors induced by the calibration procedure (i.e., an error of ∼10%). Unfortunately, we were unable to estimate the errors in the flow recovery because the pump flow in the phantom does not easily translate to a true blood flow index in the phantom. We did show, however, that the recovered $F_{1}$ values were independent of changes in the pump flow and changes in the absorption of the second layer and that the recovered $F_{2}$ values remained stable during independent changes in $\mu_{a,2}$. When comparing our results at the lowest pump setting (i.e., 1.5  L/min), we obtained very similar results for $F_{1},F_{2}$, and $F_{\mathrm{SI}}$ (median [IQR]): $F_{1}=1.47[1.43,1.5]\times 10^{-8}\;{\mathrm{cm}}^{2}/s$, $F_{2}=1.79[1.42,2.1]\times 10^{-8}\;{\mathrm{cm}}^{2}/s$, and $F_{SI}=1.66[1.61,1.73]\times 10^{-8}\;{\mathrm{cm}}^{2}/s$. Although this is an imperfect comparison, we think this supports the accuracy of our model in a situation in which $F_{1}\approx F_{2}$.

The simulation results discussed above were obtained from data generated in 2L geometries (i.e., CSF, gray matter, and white matter were merged into one homogeneous tissue type; scalp and skull were merged into a second homogeneous tissue type). The treatment of the adult head as a 2L medium is a major simplification. Although the concatenation of the scalp and skull into one homogeneous tissue type roughly mimics the case wherein a high probe pressure occludes the scalp flow, the scalp and skull flow levels are typically quite different.^17^^,^^27^^,^^28^ Our pilot simulations show that when the skull flow is much lower than the scalp flow, the performance of the constrained 2L fitting algorithm substantially worsens but still exceeds that of the SI algorithm (Appendix C). This finding motivates the use of more complex head models for quantitative accuracy. The effects of CSF on the recovered results also warrants further investigation, as there is mixed evidence in the literature of its importance on the recovered CBF.^20^^,^^67^

In summary, we used high-fidelity simulations of FD-DOS and DCS data at commonly used source–detector distances to demonstrate that a constrained 2L approach improves the accuracy of cerebral measurements compared with the conventional SI approach. Importantly, we observed that the numerical stability of the reconstructions with the constrained 2L and SI approaches were comparable. One of the constraints used is homogeneous tissue reduced scattering, which is necessary because the FD-DOS signals are minimally influenced by the cerebral tissue reduced scattering coefficient (at source–detector separations up to 5 cm). Compared with cerebral absorption, the recovery of CBF was less sensitive to inhomogeneous tissue scattering but more sensitive to errors in the extracerebral layer thickness. The impact of extracerebral layer thickness errors on FD-DOS/DCS measurements can be mitigated with future strategies that boost their brain sensitivity.

## 5 Appendix A: Solutions of Photon Diffusion Model to Semi-Infinite and Two-Layer Geometries

In diffusive media (such as biological tissue), the light transport can be modeled with a photon diffusion equation, which is written (in the frequency-domain approach) as

$$(\nabla^{2}-K_{0}^{2}(\omega ))\Phi (\overset{\to }{r},\omega )=-S(\overset{\to }{r},\omega ),$$ (5)

where $K_{0}^{2}(\omega )=(v\mu_{a}+i\omega )/D$; $D=v/(3(\mu_{a}+\mu_{s}^{\prime }))$ is the diffusion coefficient, $\mu_{a}$ and $\mu_{s}^{\prime }$ are the absorption and reduced scattering coefficients, respectively, v is the speed of light in the medium, $\Phi (\overset{\to }{r},t)$ is the fluence rate at the position $\vec{r}$, ω is the frequency of modulation of the light source, and $S(\vec{r},\omega )$ is a source term. The diffusion equation was previously solved for many different geometries, including homogeneous SI media and 2L media.^1^^,^^23^ Below, we quickly show the solutions for SI and 2L media.

### 5.1 Semi-Infinite Media

To obtain the solution for a homogenous SI media, we consider the source, $S(\vec{r},\omega )$ to be a punctual source located at $z_{0}={\left(\mu_{a}+\mu_{s}^{\prime }\right)}^{-1}$.^1^^,^^3^ Using the extrapolated zero boundary condition, we arrive at the SI solution as:

$$\Phi (\overset{\to }{r},\omega )=\frac{v}{4\pi D}[\frac{e^{-K_{0}r_{1}}}{r_{1}}-\frac{e^{-K_{0}r_{2}}}{r_{2}}].$$ (6)

Here $r_{1}=\sqrt{(z-z_{0}{)}^{2}+\rho^{2}}$ and $r_{2}=\sqrt{(z+2z_{b}+z_{0}{)}^{2}+\rho^{2}}$, and $z_{b}$ is defined as

$$z_{b}=\frac{2}{3(\mu_{a}+\mu_{s}^{\prime })}\frac{1+R_{\mathrm{eff}}}{1-R_{\mathrm{eff}}},$$ (7)

where $R_{\mathrm{eff}}$ is the fraction of photons that are internally diffusely reflected at the medium boundary. The DCS solution has an identical form as the FD-DOS solution from Eq. (6), with the difference being that $K_{0}$ is replaced with $K^{2}(\tau )=(v\mu_{a}+2v\mu_{s}^{\prime }k_{0}^{2}F_{\mathrm{SI}}\tau )/D$, where $k_{0}=2\pi /\lambda$, $F_{\mathrm{SI}}$ is the flow index, and τ is the delay time of the autocorrelation function.

### 5.2 Two-Layer Media

In this study, we opted to use the solution of the photon diffusion equation for a 2L cylinder as it is more computationally robust than the standard 2L solution for a laterally unbounded medium.^16^^,^^23^ Specifically, we modeled the tissue as an infinitely thick cylinder with radius a, composed of two layers: the first layer, with thickness ℓ, represents the extracerebral tissues; the second layer is infinitely thick and means the cerebral tissues [Fig. 1(a)]. Although we restricted our discussion to the FD-DOS and CW-DCS solutions, our results can be extended to time-domain measurements by applying a Fourier-transform to the solution presented below.

By solving the FD-DOS diffusion equation [Eq. (5) for a 2L cylinder, it is possible to show that the fluence in the k’th layer can be written as^16^^,^^23^

$$\Phi_{k}(\overset{\to }{r},\omega )=\frac{1}{\pi a^{\prime 2}}\overset{\infty }{\underset{n=1}{\sum }}G_{k}(s_{n},z,\omega )J_{0}(s_{n}\rho )J_{1}^{-2}(a^{\prime }s_{n}),$$ (8)

where $J_{n}$ are the Bessel functions of first kind and order n and $s_{n}$ are the positive roots of the zero-order Bessel function of the first kind divided by $a^{\prime }=a+z_{b}$ (i.e., $J_{0}(a^{\prime }s_{n})=0$). Here $z_{b}$ is identical to Eq. (7). Because we use the reflectance geometry in cerebral applications of diffuse optics, our main interest is the fluence at the first layer $\Phi_{1}$. Note that here we are assuming that both layers have the same index of refraction (i.e., $n_{1}=n_{2}$) and that our source is located on the center of the cylinder. For this case, $G_{1}$ is defined as

$$G_{1}(s_{n},z,\omega )=\frac{\exp(-\alpha_{1}|z-z_{0}|)-\exp(-\alpha_{1}(z+z_{0}+2z_{b}))}{2D_{1}\alpha_{1}}\;+\frac{\sinh(\alpha_{1}(z_{0}+z_{b}))\sinh(\alpha_{1}(z+z_{b}))}{D_{1}\alpha_{1}\;\exp(\alpha_{1}(\ell +z_{b}))}\;\times \frac{D_{1}\alpha_{1}-D_{2}\alpha_{2}}{D_{1}\alpha_{1}\;\cosh(\alpha_{1}(\ell +z_{b}))+D_{2}\alpha_{2}\;\cosh(\alpha_{1}(\ell +z_{b}))},$$ (9)

where $\alpha_{k}=\sqrt{\frac{v\mu_{a,k}}{D_{k}}+s_{n}^{2}+\frac{i\omega }{D_{k}}}$ and $z_{0}={\left(\mu_{a}+\mu_{s}^{\prime }\right)}^{-1}$. To compute the diffusely reflected intensity (R), we use Fick’s law^16^ as

$$R\;(\overset{\to }{r})=\;{D_{1}\frac{\partial \Phi_{1}\;(\overset{\to }{r},\;\omega )}{\partial z}|}_{z=0}.$$ (10)

Finally, the theoretical amplitude (${\mathrm{AC}}_{\mathrm{theo}}$) and phase ($\theta_{\mathrm{theo}}$) from the diffusely reflected intensity is given as

$${\mathrm{AC}}_{\mathrm{theo}}=|R(\overset{\to }{r})|,$$ (11)

$$\theta_{\mathrm{theo}}=-\arg[R(\overset{\to }{r})].$$ (12)

The diffusion equation for DCS is formally identical to Eq. (5) but with a different wave vector in which $K_{0}\to K(\tau )=v(\mu_{a}+2\mu_{s}^{\prime }k_{0}^{2}F\tau )/D\;$, where $k_{0}=2\pi /\lambda$, F is the flow index and τ is the delay time of the autocorrelation function. Based on the similarity between FD-DOS and DCS, we show that the DCS solution for a 2L infinitely thick cylinder is formally identical to the FD-DOS case [Eqs. (8) and (9)] but with a different $\alpha_{k}$:^1^^,^^68^

$$\alpha_{k}=\sqrt{\frac{v\mu_{a,k}}{D_{k}}+s_{n}^{2}+2\frac{v\mu_{s,k}^{\prime }k_{0}^{2}F_{k}\tau }{D_{k}}},$$ (13)

where $F_{k}$ is the flow index in the k’th layer. Note that the DCS solution depends on the optical properties ($\mu_{a,1}$, $\mu_{a,2}$, $\mu_{s,1}^{\prime }$, and $\mu_{s,2}^{\prime }$) as well as the flow index ($F_{1}$ and $F_{2}$) of each layer.

## 6 Appendix B. Example fits for FD-DOS and DCS

**Fig. 12:**
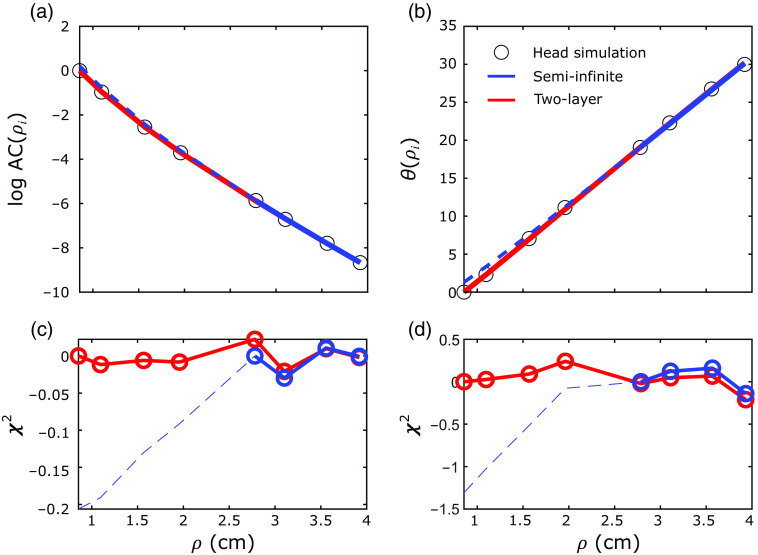
Example fits for FD-DOS data from the realistic head geometry with $\mu_{a,1}=0.1{\;\mathrm{cm}}^{-1}$, $\mu_{a,2}=0.15{\;\mathrm{cm}}^{-1}$, and $\mu_{s,1}^{\prime }=\mu_{s,2}^{\prime }=10{\;\mathrm{cm}}^{-1}$. Logarithm of (a) the amplitude ($\log\;\mathrm{AC}(\rho_{i})$) and the (b) phase ($\theta (\rho_{i})$) are plotted versus the source–detector separation for the simulated data (black circles), as well as the recovered fits from the 2L (red lines) and SI homogeneous (blue lines) approaches. We also plot the residuals ($\chi^{2}$) for (c) amplitude and (d) phase fits for the 2L (red lines) and SI (blue lines) approaches at each source–detector separation. Importantly, for the SI homogeneous fits, we only used data from the longer source–detector separations (i.e., ρ=2.8, 3.2, 3.6, and 4.0 cm).

**Fig. 13:**
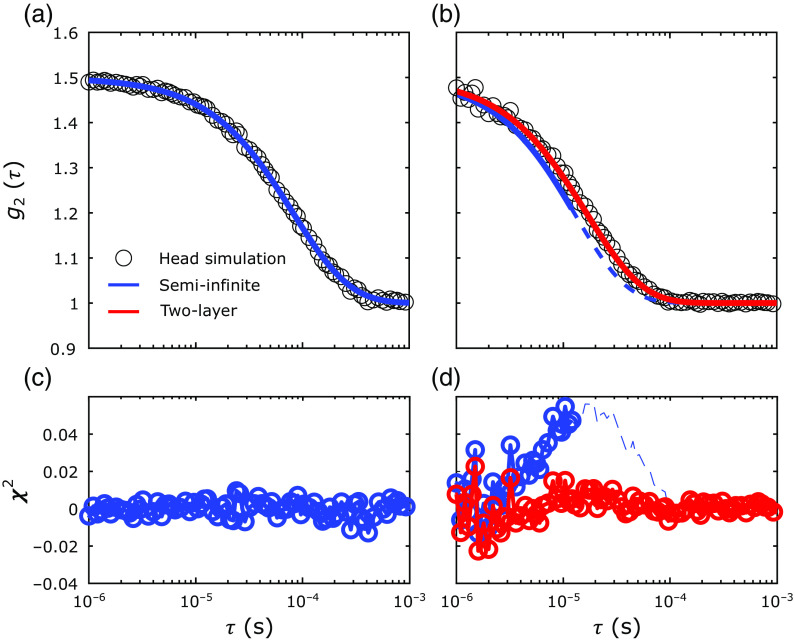
Example fits for DCS data from the realistic head geometry with $\mu_{a,1}=0.1{\;\mathrm{cm}}^{-1}$, $\mu_{a,2}=0.15{\;\mathrm{cm}}^{-1}$, $\mu_{s,1}^{\prime }=\mu_{s,2}^{\prime }=10{\;\mathrm{cm}}^{-1}$, $F_{1}=10^{-8}\;{\mathrm{cm}}^{2}/s$, and $F_{2}=8\times 10^{-8}\;{\mathrm{cm}}^{2}/s$. The autocorrelation curves ($g_{2}(\tau )$) at the short [(a) ρ=0.8  cm] and long [(b) ρ=2.5  cm] source–detector separations are plotted versus the delay times (τ) for the simulated data (black circles), as well as the recovered fits from the 2L (red lines) and SI homogeneous (blue lines) approaches. We also plot the residuals ($\chi^{2}$) for the (c) short and (d) long source–detector separations fits for the 2L (red lines) and SI (blue lines) approaches at each delay time. Importantly, for the SI homogeneous fits, we only used data from the longer source–detector separations (i.e., ρ=2.8, 3.2, 3.6, and 4.0 cm).

In this appendix, we present illustrative fits of ${AC}_{meas}$ and $\theta_{meas}$ and the respective fit residuals ($\chi^{2}$) for the head simulations in Fig. 12. We also present the illustrative fits and the fit residuals for the DCS intensity autocorrelations functions in Fig. 13.

## 7 Appendix C. NIRFASTer Simulations in a Three-Layered Realistic Head Geometry

**Fig. 14:**
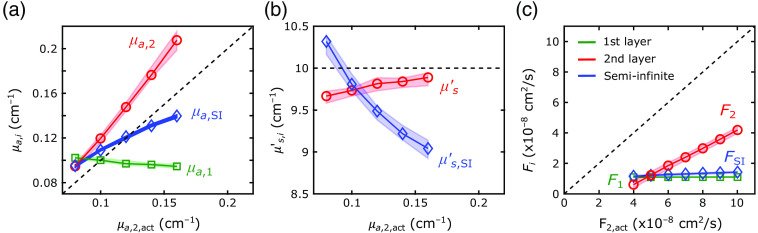
Recovered optical properties and flow indices for a three-layer realistic head simulation. The (a) recovered absorption coefficient [$\mu_{a,i}$] and (b) reduced scattering coefficients [$\mu_{s,i}^{\prime }$] are plotted against the actual second-layer values (the second-layer values were estimated based on a homogeneous phantom measurement using the same volume ratio of ink and intralipid). (c) The recovered flow indices ($F_{i}$) are plotted against the pump flow setting used in the experiment. In all cases, the green lines represent the absorption coefficient ($\mu_{a,1}$) and flow index ($F_{1}$) for the first layer, and the red lines represent the values for the second layer (i.e., $\mu_{a,2}$, $\mu_{s}^{\prime }$, and $F_{2}$, respectively). Blue lines refer to the values recovered using a homogeneous SI model (i.e., $\mu_{a,\mathrm{SI}},\mu_{s,\mathrm{SI}}^{\prime }$, and $F_{\mathrm{SI}}$). In all cases, each point denotes the medians of the recovered values across all simulations (with varying either pump flows or second-layer absorption); shaded areas represent the IQR.

On Fig. 14, we present the results obtained with our proposed two layer algorithm when fitting data from a simulation using a three-layered realistic head geometry. Although we obtained a worse accuracy compared to the two-layered head geometry, our proposed two-layer algorithm was still superior to the traditional homogeneous SI approximation.

## Code, Data, and Materials Availability

The data and code presented in this study are available on request from the corresponding author.
